# Navigating promise and perils: applying artificial intelligence to the perinatal mental health care cascade

**DOI:** 10.1038/s44401-025-00030-7

**Published:** 2025-07-23

**Authors:** Karlene Cunningham, Valentina Mărginean, Ray Hylock

**Affiliations:** 1 Brody School of Medicine at East Carolina University, Greenville, NC USA; 2 College of Allied Health Sciences, East Carolina University, Greenville, NC USA

**Keywords:** Psychology, Information systems and information technology, Health care

## Abstract

The perinatal mental health care cascade is wrought with systemic issues contributing to under-detection and outcome disparities. Herein, we examine its unique characteristics and explore how artificial intelligence (AI) may improve care while acknowledging associated ethical considerations and implementation challenges. We emphasize the need for policy reforms to screening, data collection, and regulatory processes to build ethical and robust AI-enhanced health system infrastructures.

## Introduction

Perinatal mental health conditions (PMHCs) are the most prevalent yet underdiagnosed complication of pregnancy and postpartum^1,2^. PMHCs include conditions such as depressive/bipolar disorders, anxiety disorders, obsessive-compulsive disorder, posttraumatic stress disorder, substance use disorders, and psychosis. The estimated societal financial cost of untreated PMHCs is a staggering $14.2 billion^3^. This is an underestimation as it excludes the often-overlooked mental health experiences of non-birthing partners^4^. The most disturbing impact is the loss of life, as the leading causes of postpartum-period deaths are suicide and overdose^5–7^. PMHC-related deaths are preventable with appropriate identification, timely intervention, and adequate treatment. However, due to systemic shortcomings, the current healthcare infrastructure is not built to support birthing people and their families, especially those from minoritized backgrounds^8,9^.

Artificial intelligence (AI) is a powerful application of computer science in which computers can perform cognitive functions such as thinking, learning, decision-making, and problem-solving. The recent innovative application of large language models (LLMs) and generative AI has catalyzed significant innovation and task automation across sectors, including healthcare^10^. The application of AI to perinatal mental health is in its infancy but shows potential to address gaps in the current treatment cascade. This paper aims to provide an overview of AI applications that address current barriers in the perinatal mental health care cascade while highlighting possible perils of such applications, specifically related to equity and safety concerns. This work adds to existing literature by applying the treatment cascade framework to bridge AI technical capabilities and clinical perinatal mental health needs.

## Applying AI to the perinatal mental health care cascade

**Fig. 1:**
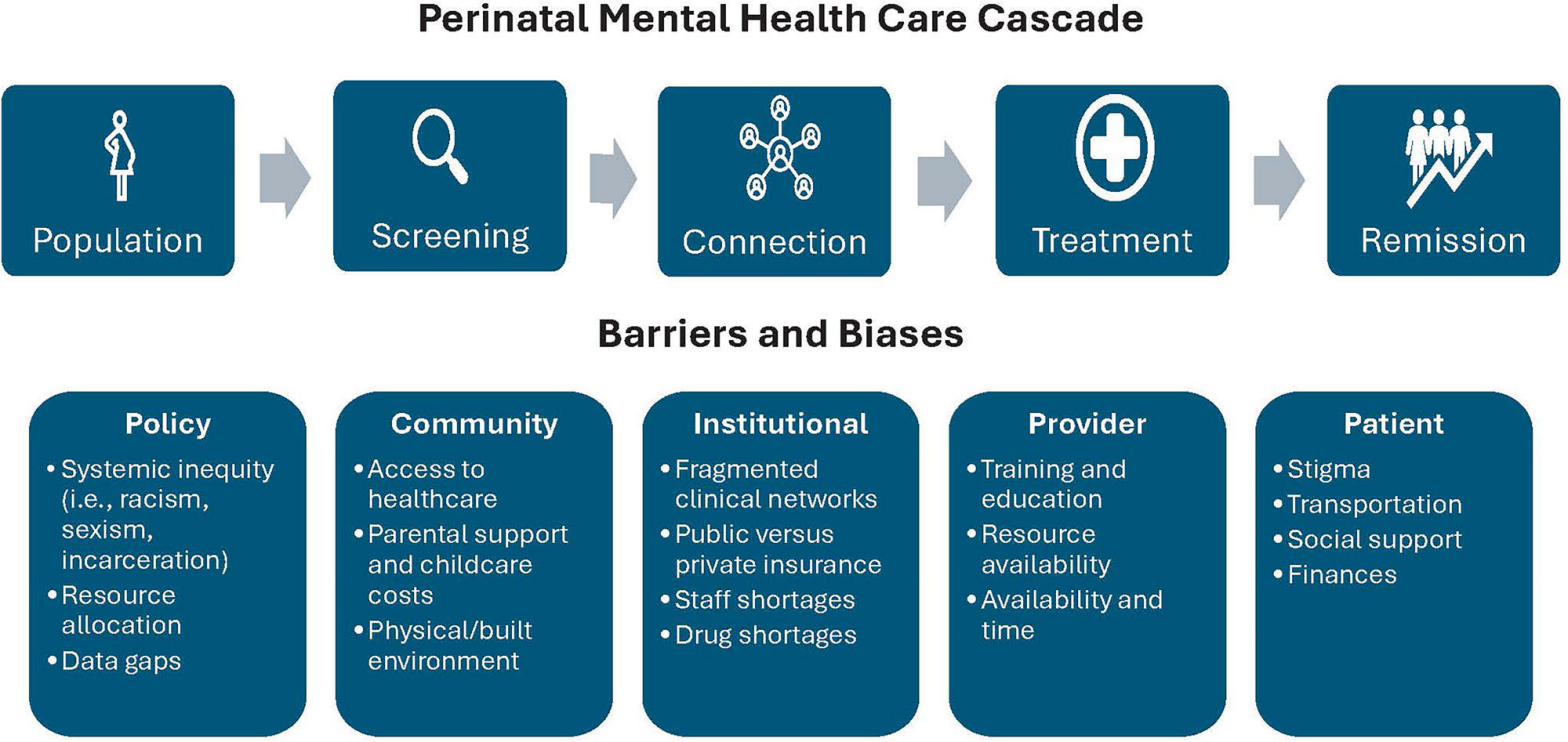
Perinatal mental health care cascade and associated barriers. This figure illustrates the sequential stages of perinatal mental health care delivery and the multilevel barriers that can impede progression through the care continuum. The cascade represents the ideal pathway from initial population screening through achievement of symptom remission, with each stage representing a transition point where patients may be lost to follow-up or experience delays in care. The care cascade consists of four primary stages: (1) Screening & Identification - systematic screening of the perinatal population using validated instruments to identify individuals at risk for or experiencing mental health conditions; (2) Connection to Specialized Care - successful referral and linkage to appropriate mental health services, including initial appointment attendance; (3) Treatment Delivery - provision of evidence-based interventions tailored to perinatal mental health needs; and (4) Remission - achievement of clinically significant symptom reduction and functional improvement. Barriers to care progression are categorized into socioecological domains. Patient-level barriers comprise factors such as mental health stigma, limited social support, and transportation. Provider-level barriers encompass healthcare provider factors, including training and education, resource availability, time constraints, symptom acceptability, and effectiveness. Institutional-level barriers include fragmented clinical networks, services available based on insurance type, staffing levels, and drug shortages. Community barriers encompass system-level obstacles, including the cost of care and support, as well as limited access to specialized providers. Policy-level barriers include systemic inequities, resource allocation, and data gaps.

The cascade of care (Fig. 1) is a theoretical framework outlining care stages associated with quality care and used to monitor a healthcare system’s effectiveness in addressing care needs^11^. Within perinatal mental health, this cascade has been estimated for perinatal depression because it is the most screened and researched PMHC^8^.

### Population

Approximately 5 million pregnancies occur in the US annually, resulting in roughly 3.6 million live births^12^. Current estimates suggest 20% of these pregnancies will have PMHC complications, with an increased risk for those with pregnancy complications, a neonatal intensive care stay, or experiencing a loss^1^. For minoritized populations, this estimate grows to nearly 50%^13^, largely driven by systemic exposures to factors (e.g., systemic racism, trauma) that increase the chance of experiencing mental distress and drive limited connection to adequate support^9,14^. These same systemic drives also limit the availability of PMHC data for these marginalized communities.

### Screening

The current American College of Obstetricians and Gynecologists (ACOG) guidelines for pregnancy management recommend screening for depression and anxiety at least once during prenatal visits (i.e., 10-15 recommended visits) and at least once in the postpartum period if screening was performed during the prenatal period^15,16^. They also urge the inclusion of comprehensive assessments of physical, social, and psychological well-being during postpartum encounters^15,16^. These guidelines have recently evolved to highlight the increasing need to screen for PMHCs; however, current clinical practice continues to lag^17^. Despite the growing recognition of PMHCs and the specific awareness of perinatal depression, current estimates hold that up to 75% of clinically significant perinatal depression remains unidentified^14^. Disparities persist for minoritized groups, with Black, Asian, and Indigenous birthing people being consistently less likely to receive PMHC screenings^9^. Such inequality appears to be a significant driver of overall inequity in perinatal depression and other PMHCs. Given the relatively low screening of other PMHCs, detection rates are presumed to be worse, further exacerbating treatment discrepancies^17,18^.

Data about non-birthing partners is scarce but growing. Estimates suggest that approximately 10% of non-birthing partners experience perinatal depression or anxiety^19,20^. This is likely an underestimate due to the unique presentation of depressive symptoms often observed among cis-male partners, with some studies suggesting an equal representation of depression among cis-male partners when these differences are considered^19^. Similarly, current estimates of postpartum deaths do not include those occurring among fathers and co-parents, as researchers are only now recognizing the risk of mental health conditions within this population^19,21^. To our knowledge, no data is available specifically for rates of under-identification of mental health needs for non-birthing partners. Currently, there are no standardized recommendations to screen non-birthing partners regularly during the pregnancy period^22^.

#### Predictive modeling for early detection

While current identification relies on self-administered screening tools and provider assessment, machine learning—a specific type of AI—offers novel approaches to risk prediction. A recent scoping review of 14 articles examining the application of AI in PMHC research found that approximately 60% were focused on developing risk prediction models using supervised machine learning^23^. This technique uses historical data with known outcomes to forecast future cases. These predictive models were primarily developed using electronic health record (EHR) data, such as lab values, demographic variables, mental health history, and utilization, with a demonstrated acceptable discrimination ability (i.e., AUC > 0.7)^23^. Still, achieving “good” performance (i.e., AUC > 0.8) typically required incorporating PMHC screening data or historical mental health data, suggesting standard EHR data alone may be insufficient for optimal risk detection^24,25^. While EHRs contain both structured (e.g., values) and unstructured data (e.g., clinical notes), these models only analyze structured elements, excluding valuable information from clinical notes. Unstructured data has historically been challenging to analyze because meaningful and consistent insights cannot be extracted without the use of computationally intensive natural language processing (NLP) methods^23,26^.

Two notable studies from this review demonstrated novel NLP applications. One successfully scanned clinical notes for risk indicators with acceptable recall and precision^27^. Another study analyzed approximately 67,000 social media posts to assess paternal perinatal depression risk using NLP and classification, a supervised learning technique that predicts membership in a class (e.g., low, moderate, or high risk)^28^. This application identified high-risk fathers based on their linguistic patterns and engagement levels. A similar study focused on Reddit posts by pregnant women leveraged sentiment analysis—a method used to analyze text data to detect emotional states—similarly finding the ability to detect and track changes in depressive sentiments over time^29^.

Another application of AI language processing that relies heavily on deep learning—a type of machine learning built on artificial neural networks—is ambient AI scribes. These AI scribes automatically convert patient-provider conversations into documentation while allowing clinicians to focus on patient care^30^. Beyond documentation, these systems can analyze conversation patterns to identify language indicating psychological distress. Pilot trials examining voice-based models for perinatal depression showed higher sensitivity and recall, signaling this approach may be an improvement over traditional screening method. Additionally, when integrated with computer vision technology that analyzes facial expressions, these multi-modal systems may detect subtle indicators of PMHCs that routine screening might miss^31^. This approach aligns with patient preferences, as many individuals feel more comfortable discussing their difficulties directly with clinicians than completing standardized screening tools^32^. Although speech and visual diagnostics are still emerging, they offer the potential to facilitate more natural communication between patients and providers regarding PMHC concerns. Overall, leveraging these applications to improve identification and risk stratification are promising for overcoming traditional identification barriers.

### Connection to care and treatment initiation

Beyond identifying those at risk, connecting patients to the appropriate level of care is often challenging, as mental healthcare resources are fragmented in most communities. Patient preferences for care and their match to available resources also prevents appropriate support for managing PMHCs. Primary care providers (PCPs) and perinatal providers, such as obstetricians/gynecologists and midwives, are patients’ most common clinical touchpoints. However, few perinatal health specialists feel comfortable managing the full spectrum of PMHC, oftentimes due to a lack of formal training in behavioral medicine^33^. As such, adequate management of PMHCs typically requires referrals to behavioral medicine specialists, due to the limited number of behavioral providers integrated into perinatal clinics^33,34^. Yet, even with referrals, significant workforce shortages limit access to these services, with many waiting months to be connected with the appropriate provider. Currently, 49% of people in the US live in mental health shortage areas, with less than 30% of psychiatric health needs being met nationwide^35^. The shortage is particularly acute for PMHC-trained psychiatrists, as 21 states have fewer than one perinatal psychiatrist per 5000 births^34^. Most states require dozens to hundreds of additional psychiatrists to meet a growing demand^34^.

Once connected, there are several evidence-based treatments available for PMHCs, including medication management, psychotherapy modalities, and community-based care support. Cognitive behavioral therapy, interpersonal psychotherapy, and other psychotherapy modalities serve as first-line treatments for mild to moderate PMHCs^36^. Patients generally prefer psychotherapy and community-based support, but access varies significantly^36,37^. Frequently cited initiation rates range from 13% to 15%, although well-resourced integrated systems have demonstrated rates up to 60%^8,38^. Most healthcare systems struggle to provide effective, tailored psychotherapy due to mental health workforce shortages, restrictive insurance coverage, and inadequate reimbursement for scalable delivery methods. Transportation and childcare needs can also impact the continuation of treatment, often necessitating multiple visits per month during the acute phase of treatment. Most mental health outpatient offices lack childcare support, or if children are allowed to come, they may not be built with their needs in mind^39^.

Treatment for severe PMHCs requires combined medication management and psychotherapy for optimal outcomes^40^. Several established pharmacological options exist, including selective serotonin reuptake inhibitors, serotonin and norepinephrine reuptake inhibitors, and atypical antidepressants, with mood stabilizers and antipsychotics used in specific cases^41^. Recently FDA-approved neuroactive steroids for severe postpartum depression face adoption barriers due to administration challenges, side effect concerns, and limited provider awareness. Perinatal providers often restrict their medication management to familiar PMHCs (e.g., depression), showing reluctance to modify failed treatment regimens or manage complex conditions like bipolar disorder and psychosis^42^. Drug shortages have also impacted conditions such as ADHD, where stable patients required different agents due to supply issues^43,44^. The PMHC-trained psychiatrist shortage, combined with perinatal provider management reluctance, continues to be a significant barrier to appropriate medication access, especially to novel agents^40,41^.

Peer support specialist, individuals with lived experiences of PMHC with or without additional training, offers a valuable alternative for individuals who feel stigmatized by potential PMHC diagnoses—a common experience during the perinatal period^45,46^. Drawing from their PMHC experiences, peer support workers provide understanding and mutual empowerment to those navigating similar challenges. These services are usually provided in the community through local and national organizations. Similarly, as non-clinical professionals, doulas offer physical, informational, and emotional support throughout the perinatal period^47^. They help mitigate factors that may worsen PMHCs and can serve as a bridge to professional resources^45^. In addition, doula support has been shown to lower PMHC incidence and reduce exposure to birth trauma^47^. Connection to a doula or peer support specialist is an often underutilized option due to a lack of awareness about these professionals, limited insurance coverage, and the cost-prohibitive nature of pursuing credentialing to receive payment from insurance when programs are available. Doulas are generally more accessible to well-resourced individuals who can pay privately and have had fewer life experiences that could increase the risk of PMHCs, despite studies suggesting that doulas are most effective for those who are resource-limited and at higher risk^48^. The increased use of these community-based providers has improved outcomes for marginalized groups less likely to be maintained in the cascade.

#### Leveraging natural language for connection and treatment

The enhanced capabilities of AI applications to analyze and generate language have significantly improved their usefulness as assistants in clinical interactions with patients. This will be particularly helpful in addressing knowledge gaps for providers without PMHC expertise. Traditional voice assistants (e.g., Siri, Alexa) provided clinically accurate PMHCs information only 14–29% of the time; however, newer LLM-based systems (e.g., ChatGPT) achieved accuracy rates of 79–100% on standardized clinical questions^49,50^. Increasing access to LLM-enhanced voice assistance will provide an opportunity for the dissemination of accurate PMHC treatment recommendations, thereby improving medication management in areas with prescriber shortages^51^. State-level perinatal psychiatric call lines have demonstrated that increasing providers’ knowledge through access to a PHMC-trained professional improves the management of PMHCs in their clinics using local resources^52^. Despite their effectiveness, perinatal psychiatric consultation lines are only available in 25 states, and provider awareness of the national consultation service remains limited^52^. The long-term sustainability of these support services is further challenged by their dependence on grants and governmental funding. If appropriately trained and updated, healthcare-specific voice assistance supported by secure LLMs could provide a scalable solution for improved care when a PMHC is identified.

**Fig. 2:**
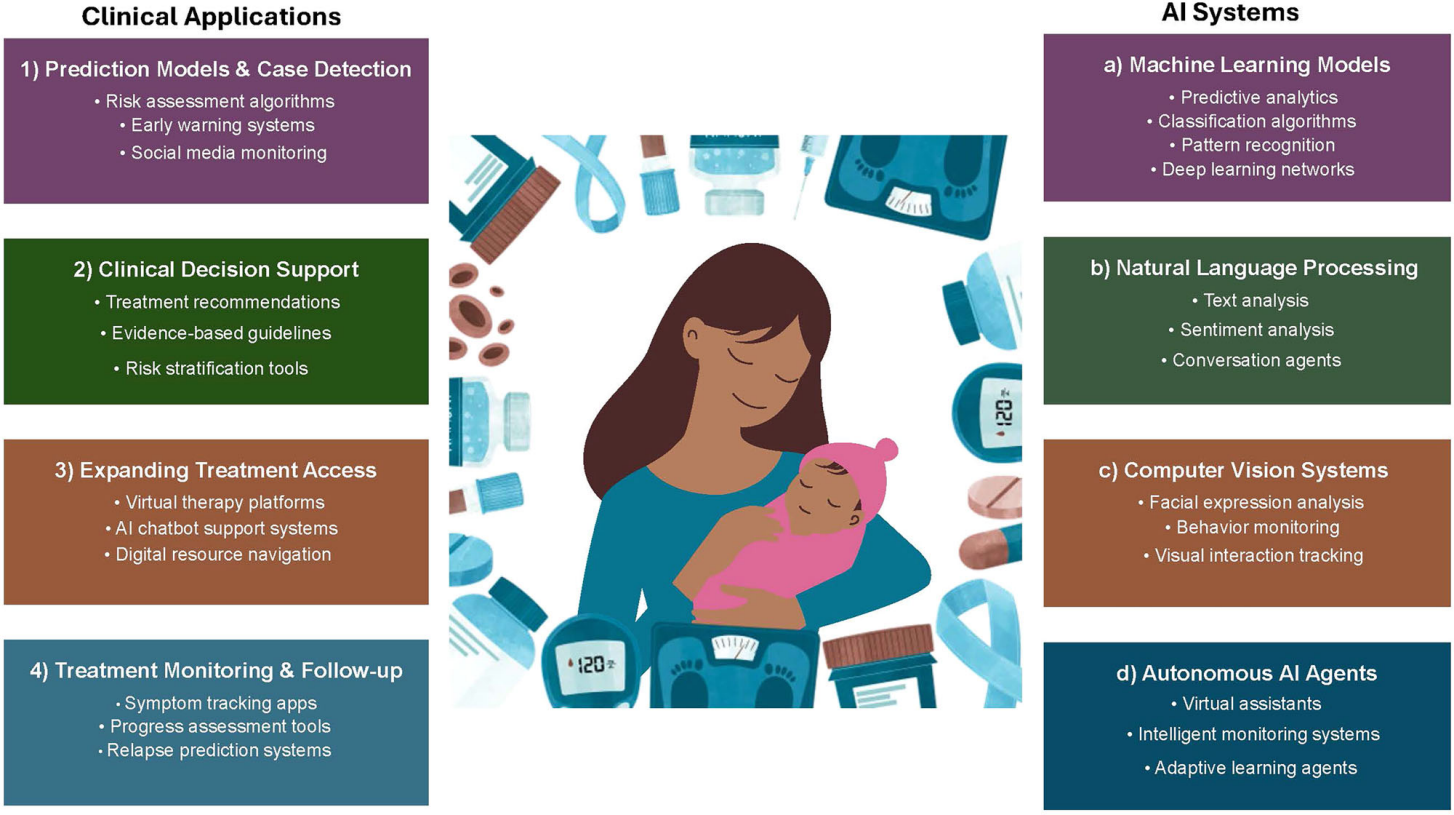
Potential AI applications across the PMHC care cascade. This figure illustrates the various AI applications currently deployed or that could be deployed to enhance the PMHC treatment cascade, as well as the AI systems that underpin these technologies. Clinical applications include four key domains: (1) prediction models and case detection, encompassing risk stratification, early warning systems, and social media monitoring; (2) clinical decision support, providing treatment recommendations, evidence-based guidelines and risk stratification tools; (3) treatment access expansion through teletherapy platforms, AI chatbot support systems, and digital resource navigation; and (4) treatment monitoring via symptom tracking apps, progress assessment tools, and relapse prevention systems. The clinical applications leverage four primary AI technology categories: (a) machine learning, incorporating predictive analytics, classification algorithms, pattern recognition and deep learning; (b) natural language processing enabling text analysis, sentiment analysis, and conversation agents; (c) computer vision systems that include facial expression analysis, behavioral monitoring, and visual interaction tracking; and (d) autonomous AI agents such as virtual assistants, intelligent monitoring systems and adaptive learning agents.

AI-based systems can also extend PMHC connection and treatment beyond clinical interactions through autonomous AI agent tools, such as chatbots^53^. These tools can engage patients, provide basic emotional support, facilitate psychotherapy, and connect users to crisis resources—functions that mirror community-based support, such as doulas and peer specialists. When integrated with EHRs, these systems would enable clinicians to monitor and respond to care cascade initiation or escalation needs. Although digital mental health solutions are not new, over 90% of traditional psychotherapy chatbots rely on rule-based models with predetermined response sets^54^. However, emerging AI technologies enable more flexible and responsive interactions. Perinatal populations report being open to using mental health chatbots. Current AI-powered apps have demonstrated the ability to reduce depression symptoms and have provided safeguards for privacy^53,54^. Similarly, chatbots have been developed outside of the mental health space to provide doula-like informational and emotional support. These informational chatbots also combine connection features to connect users with local doulas to provide labor and deliver support^55,56^. This expanded access to resources may help reduce perinatal access disparities (Fig. 2).

### Achieving remission

Treatment adherence is crucial for symptom remission for PMHCs. Remission is defined as the absence of symptoms of the disorder at a clinically diagnosable level and often requires continued maintenance treatment. Medication management during pregnancy faces particularly high discontinuation rates—up to 80%—largely due to concerns about fetal effects, despite evidence that untreated PMHCs pose similar risks^57^. Discontinuation rates are generally lower for psychotherapy but increase postpartum, a specifically vulnerable period. Common barriers to treatment continuation include time constraints, financial costs, and poor provider fit. PMHC treatments are highly effective, with pooled estimates suggesting that over 50% of individuals achieve remission with adequate treatment^58,59^. Despite these effective treatments, estimates suggest only 3.2% to 4.8% of people with perinatal depression will ultimately achieve remission^8^.

#### AI-enhanced treatment monitoring and outcome detection

Many of the AI-based treatment enhancements may also help to reduce premature discontinuation of treatment. For example, the integration of AI facilitated psychotherapy applications with traditional therapy, especially those provided virtually, addresses access, transportation, and time barriers, which are often cited as reasons for discontinuing some psychotherapy interventions^60^. Autonomous AI agents can also work to re-engage patients, in medication management or therapy, by adapting their approach based on previous interactions with the patient. This adaptive learning is another feature that is enhanced through the flexibility of generative systems compared to prior rule-based frameworks. In addition, early work suggests the ability to integrate commercial wearable device information not only to detect postpartum depression but also to monitor progress^61^. AI tools have also demonstrated potential as an early detection tool for those who will experience persistent symptoms or relapse versus those who are more likely to achieve remission^62^. Those with persistent symptoms are more likely to require different agent trials or a combination of treatment modalities, so knowing this risk potential would allow for more tailored treatment by providers.

## The possible perils of AI for perinatal mental health cascade

**Fig. 3:**
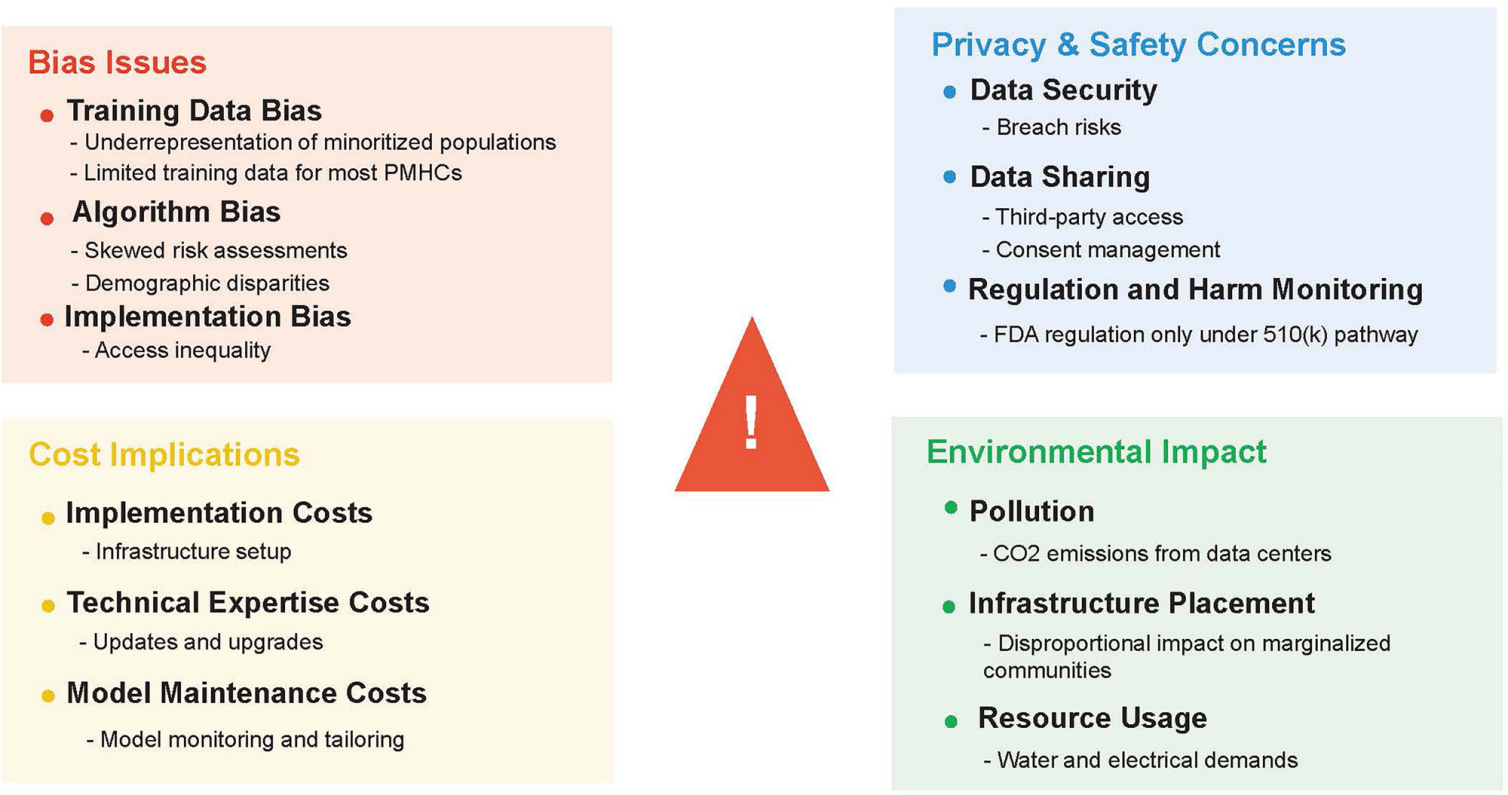
Potential pitfalls in AI implementation for perinatal mental health. This figure outlines some of the potential pitfalls of AI implementation across four domains: Bias, including due to the underrepresentation of minoritized groups in training data, limited structured information related to PMHCs outside of depression and anxiety, disparities in screening data resulting in skewed risk assessments, and access inequity in the rollout of systems. Cost implications, including initial set-up costs, employing technical expertise needed for updates, and the cost incurred to monitor models and tailor them to the institution. Privacy and safety concerns outline the risks of breaches, data sharing, consent, and regulatory considerations. Environmental impact encompasses concerns about pollution, the placement of AI infrastructure in marginalized communities, and the natural resources demands required to sustain these systems.

The potential of AI to expand access to perinatal mental healthcare is considerable; however, important technical, ethical, and practical challenges must be addressed before its full potential is realized (Fig. 3). Improvement will require addressing several systemic drivers of inequity and adopting a multifaceted approach that involves technology, policy reform, and stakeholder engagement to ensure equitable outcomes.

### Flawed foundations

While AI offers significant potential to enhance access to perinatal mental healthcare, its effectiveness is hindered by critical limitations in training data, which often perpetuate systemic inequities^63^. Current healthcare systems collect limited screening data, primarily focusing on depression and anxiety while neglecting other PMHCs, thereby restricting the scope of AI applications. This narrow focus, compounded by disparities in data collection, compromises the accuracy and reliability of AI models, increasing the risk of algorithmic bias. Furthermore, the lack of transparency in AI training data (e.g., due to patient privacy concerns) hinders the understanding of the rationale behind clinical recommendations, thereby undermining trust among patients and providers^64^. The growing emphasis on explainable AI and algorithmic transparency further highlights these challenges, as documentation of clinical protocols and verifiable model reasoning is frequently absent^65^. Without robust bias and fairness assessments, it becomes difficult to ascertain whether developers have adequately addressed inherent data biases. This, in turn, raises concerns about the models’ efficacy and potential harm they may cause to marginalized communities^66^.

The Data to Save Moms Act represents a critical step toward addressing these issues by prioritizing perinatal data collection in the US, particularly for marginalized populations^67^. However, given documented instances of AI-driven biases adversely impacting underserved communities^68^, additional policy measures are imperative, including mandates for universal PMHC screening and requirements to demonstrate algorithmic fairness before regulatory approval. Such reforms, coupled with stakeholder collaboration, are essential to ensure AI advances promote health equity rather than exacerbate disparities^26^.

### Tailoring and model drift

Healthcare systems implementing AI solutions for perinatal mental health must carefully consider their specific patient populations and available data resources. Generic LLMs, while accessible, may not adequately address the unique needs of diverse patient populations or reflect local practice patterns and resources^69^. Effective implementation requires tailoring these tools to account for demographic variations, cultural considerations, and community-specific barriers to care that characterize each healthcare system’s patient population.

The challenge of model drift presents significant operational concerns. As clinical guidelines evolve and new research emerges, AI models become outdated, potentially leading to suboptimal or incorrect recommendations^70,71^. Healthcare systems must develop robust processes for monitoring model performance, incorporating new clinical evidence, and updating AI tools regularly. This requires sustained investment in technical expertise and infrastructure - resources many healthcare systems lack, particularly those serving underserved communities.

Persistent technological integration barriers, such as fragmented EHRs and limited interoperability between clinical systems, also hinder the effectiveness of AI tools and may introduce new risks^72^. Therefore, healthcare systems must assess their technical readiness to implement and maintain these solutions effectively over time.

### Cost and sustainability

The deployment of AI in perinatal mental health requires significant initial investments in infrastructure, expertise, and data security, posing challenges for resource-limited healthcare systems^73^. However, advancements such as cloud services, shared computing resources, and cost-effective hardware like Nvidia’s $3000 AI “supercomputer” offer promising solutions^74,75^. Despite this, sustaining AI tools will continue to be costly due to ongoing infrastructure and model maintenance, regulatory compliance, and clinical effectiveness assessments, to name a few^26^.

Beyond financial costs, AI infrastructure has substantial environmental impacts, felt more acutely in low-income communities^76^, including significant CO_2_ emissions and electrical and water demands akin to small cities. Marginalized communities disproportionately bear these environmental burdens, which can exacerbate perinatal mental health issues and widen existing health disparities. Enhancing AI model efficiency to reduce resource consumption and developing robust policies and regulations to mitigate environmental impacts are critical.

### Patient privacy and security

Developing AI models requires substantial data, raising concerns about privacy, security, and patient agency. Under the Health Insurance Portability and Accountability Act (HIPAA), organizations must notify patients of potential data uses through the Notice of Privacy Practices (45 CFR §164.520). However, if classified as a research activity, no such notification or authorization is required (45 CFR §164.512(i)). Also, outsourcing often becomes necessary, given the technical expertise and computing resources needed to develop custom LLMs. While protections are in place for research (Institutional Review Boards and Privacy Boards; 45 CFR §164.512(i)) and outsourcing (Business Associate Agreements; 45 CFR §164.502(e)) activities, neither involves the patient, diminishing their agency, which is a rising concern^77^.

AI systems are not impervious to exploitation. Breaches can compromise model performance, expose training data, and bypass implemented safety measures^78^. Healthcare systems are already battling rising cyber-attacks and resulting data breaches^79,80^, and AI systems offer an enticing target for attackers. As AI integration in healthcare expands, robust information security measures become increasingly critical.

### Patient safety and regulatory framework

Patient safety concerns regarding AI in mental health support extend beyond data security. Recent incidents involving LLM chatbots have highlighted serious risks. These include cases where AI systems provided potentially harmful mental health advice or inappropriately mimicked mental health professionals, with tragic consequences^81^. While developers have responded with enhanced safeguards and modified marketing practices, intentional design choices in AI applications present additional risks. For instance, therapy tools could potentially embed promotional content within therapeutic interactions without provider awareness^82^. These vulnerabilities underscore the critical importance of robust regulatory oversight for AI-enabled mental health technologies.

The landscape of medical device regulation for AI-enabled technologies has grown significantly, with approximately 950 FDA approvals in the past decade^83^. Most of these approvals occur through the 510(k) pathway, which evaluates moderate-risk devices by comparing them to previously approved devices, regardless of whether the predicate device incorporates AI^83,84^. This regulatory process reflects an opportunity for updates in the regulatory procedures for AI-enabled technologies through modernizing predicates and applying AI-specific safeguards^84^. In April 2024, MamaLift Plus^TM^ became the first prescription digital therapeutic authorized for the treatment of postpartum depression through the 510(k) pathway^85^. More of these technologies will be approved in the coming years. It is too soon to tell how perinatal healthcare providers will adopt these technologies, but historically, digital therapeutics have had poor uptake and integration into healthcare systems. Regulatory concerns have been a common barrier to uptake^86^.

The regulatory landscape for AI has varied between states and even more so between countries. The European Union has taken the strongest stance on attempting to regulate the broader AI community, specifically prohibiting AI systems from being developed to manipulate people, taking a risk-based approach to situations requiring regulation, and enacting penalties for companies found to be in noncompliance. Transparency and human oversight are also specifically outlined for healthcare-related applications. In contrast, in the US, the regulatory landscape is evolving through executive action^87^, as a legal framework such as the EU AI Act^88^ has yet to be established. It is still too early to tell how these different approaches to regulation will impact the development and innovation of AI applications in healthcare.

## Conclusion and future directions

As we look to the future of the perinatal mental health care cascade, change is needed to address the overwhelming gaps in connecting pregnant people and their families to adequate care. AI-enabled technologies appear to have a place in improving each step in the care cascade if we can address data fairness and quality, data security, and regulatory concerns, allowing for increased trust in these tools to not cause harm. Future research should specifically address these key concerns when developing new tools. Similarly, policy-level changes and investment in data collection quality around perinatal mental health outcomes will help support the development of better tools.

## Data Availability

No datasets were generated or analysed during the current study.
